# Exploring fractional-order new coupled Korteweg-de Vries system via improved Adomian decomposition method

**DOI:** 10.1371/journal.pone.0303426

**Published:** 2024-05-28

**Authors:** Muhammad Arshad, Saud Fahad Aldosary, Saba Batool, Irfan Hussain, Naveed Hussain

**Affiliations:** 1 Department of Mathematics and Statistics, Sub-Campus Depalpur, University of Agriculture Faisalabad, Faisalabad, Pakistan; 2 Department of Mathematics and Statistics, University of Agriculture Faisalabad, Faisalabad, Pakistan; 3 Department of Mathematics, College of Science and Humanities in Alkharj, Prince Sattam bin Abdulaziz University, Alkharj, Saudi Arabia; 4 Department of Mathematics, University of Baltistan, Skardu, Pakistan; 5 Department of Computer Science, University of Agriculture Faisalabad, Faisalabad, Pakistan; College of Mathematics and Systems Science, Shandong University of Science and Technology, CHINA

## Abstract

This paper aims to extend the applications of the projected fractional improved Adomian Decomposition method (fIADM) to the fractional order new coupled Korteweg-de Vries (cKdV) system. This technique is significantly recognized for its effectiveness in addressing nonlinearities and iteratively handling fractional derivatives. The approximate solutions of the fractional-order new cKdV system are obtained by employing the improved ADM in fractional form. These solutions play a crucial role in designing and optimizing systems in engineering applications where accurate modeling of wave phenomena is essential, including fluid dynamics, plasma physics, nonlinear optics, and other mathematical physics domains. The fractional order new cKdV system, integrating fractional calculus, enhances accuracy in modeling wave interactions compared to the classical cKdV system. Comparison with exact solutions demonstrates the high accuracy and ease of application of the projected method. This proposed technique proves influential in resolving fractional coupled systems encountered in various fields, including engineering and physics. Numerical results obtained using Mathematica software further verify and demonstrate its efficacy.

## 1 Introduction

Analytical solutions for non-linear evolution equations (NLEEs) play a crucial role in understanding nonlinear problems across various applied sciences [1–4]. The pursuit of analytical solutions for diverse NLEEs is essential, and recent literature reflects a notable effort to obtain traveling wave solutions [5–8], typically relying on variables associated with traveling waves [9, 10]. However, obtaining analytical solutions for these differential equations can be challenging, leading to the introduction of semi-analytical techniques for representation in series form. Furthermore, assessing series convergence is imperative, often involving the analysis of absolute errors when theoretical examination of convergence is feasible. Some semi-analytical methods suggest considering a limited number of terms in the series to achieve significantly improved approximations to exact solutions [11–14].

In recent decades, notable advancements have been made in exploring fractional differential equations (FDEs) and fractional calculus, carrying implications across diverse domains of applied sciences and engineering [15–17]. Fractional calculus, which addresses derivatives and integrals of non-integer order, has garnered substantial attention due to its capacity to accurately describe complex behaviors and phenomena in areas such as electromagnetic fields, acoustics, viscoelasticity, electrochemistry, cosmology, and material science [18–21]. This accuracy surpasses that achieved by traditional integer-order calculus. Given the inherent complexity of many fractional differential equations, exact solutions often remain elusive. Consequently, there has been a significant focus on developing approximate analytical solutions for FDEs, becoming a prominent area of interest in both academic research and practical applications. The application of fractional calculus has proven beneficial in modeling and control theory across a diverse array of fields [22–25].

Recently, several methodologies have emerged to address fractional differential equations. Noteworthy methods include the differential transform method [26], Meshless method [27], the ADM [28], and He’s variational iteration technique [29]. Abdulaziz applied the homotopy perturbation method (HPM) to solve systems of fractional differential equations [30], while in another study (Ref. [31]), the variational technique was employed to address systems of autonomous differential equations. Moreover, researchers have focused on solving fractional NLEEs, employing diverse techniques such as the Homotopy Analysis Method [32], VIM (Ref. [33]), He’s HPM [34], ADM [35, 36], homotopy asymptotic scheme [37], and reduced differential transform method [16]. Ren recently contributed results related to Caputo-type partial differential equations [38].

The ADM [35, 39], initially introduced by the American mathematician Adomian, has proven to be a powerful approach for approximating solutions to various nonlinear differential equations. This classical yet effective method has undergone refinements over time to address inherent limitations in the solution process and enhance result accuracy. For instance, Wazwaz in [40] introduced an innovative algorithm for computing Adomian polynomials, advancing the ADM. This modified approach effectively solved differential equations with robust non-linear terms. Another variation, based on Newton’s method, was proposed by Abbasbandy [41], introducing not only a modified ADM but also formulating numerical algorithms based on it. These contributions significantly improved the performance of the conventional ADM.

The exploration of analytical solutions for new coupled systems is crucial for understanding the characteristics of non-linear problems and soliton theory in mathematical physics and applied sciences [42, 43]. Analyzing traveling wave solutions, especially for non-linear coupled systems and higher-order NLEEs [44], provides valuable insights into the underlying physical phenomena. Lately, several systematic and potent methods have been developed to derive wave solutions of NLEEs such as modified extended algebraic method [45, 46], double (*G*′/*G*, 1/*G*)-exponential technique [47], positive quadratic technique [48] and so on [49, 50]. The investigation of solutions, structures and interactions has garnered substantial attention, resulting in diverse and meaningful outcomes [51–54].

Constructing approximate solutions for fractional order new coupled KdV system can be quite challenging and useful in various fields, particularly in the study of nonlinear wave phenomena. Approximate solutions help researchers gain insights into the behavior of these systems without having to rely solely on numerical simulations or exact analytical solutions, which may not always be feasible. Different research works have explored various forms of fractional coupled KdV equations to develop approximate solutions. The authors in [55] constructed approximate solutions for the fractional-order Korteweg-de Vries (KdV) equation. In [56], the authors focused on numerical solutions of the fractional-order coupled KdV equation with several different kernels. Additionally, the authors in [57] constructed approximate solutions for the generalized coupled KdV equation. Furthermore, in [58], the Time-Fractional Coupled KdV equation was solved using the natural decomposition technique.

In this study, we employ the projected fIADM to derive approximate solutions for the fractional order new coupled KdV system. The obtained approximate results demonstrate the effectiveness and power of this technique for fractional coupled systems. Additionally, we conduct a comparative analysis at *ρ* = 1, specifically focusing on the solutions of bright and dark solitary waves. To validate our approach and showcase its effectiveness, we perform numerical experiments using Mathematica software. To the best of our knowledge, no previous work has been reported in the literature on this fractional wave system, confirming the novelty of our results.

This manuscript is structured as follows: Section 2 provides an overview of Preliminaries. The description of the projected fADM is presented in Section 3. Section 4 describes the fractional new cKdV system. The analysis of the method for the fractional new cKdV system is detailed in Section 5. Section 6 discusses the obtained numerical results. Finally, Section 7 concludes the paper.

## 2 Preliminaries

The foundational definition of Caputo fractional-order integration and differentiation is introduced here, playing a crucial role in defining various fractional derivatives with order *ρ* > 0 [11, 16, 19, 23, 59]. The Caputo fractional derivative is particularly emphasized for its significance and relevance within this study. By embracing this fundamental definition, the research ensures a consistent and cohesive approach to the domain of fractional calculus and its practical applications. Notably, the Caputo derivative is derived from the Riemann-Liouville derivative, offering adjustments that address some of the limitations associated with the traditional Riemann-Liouville approach to fractional derivatives.

**Definition 2.1:** Supposing λ ∈ *R* and *n* ∈ *N*. A real valued function *ψ*: *R*^+^ → *R* belongs to space *C*_λ_, if ∃ *l* ∈ *R*, *l* > λ and *ψ*_1_ ∈ *C*[0, ∞) such that *ψ*(*x*) = *x*^*l*^*ψ*_1_(*x*), ∀ *x* ∈ *R*^+^. Moreover, $\psi \in C_{\lambda }^{n}$ iff *ψ*^(*n*)^ ∈ *C*_λ_.

**Definition 2.2:** The fractional derivative of *ψ*(*x*) of order *ρ* in Caputo sense is defined as

$$\begin{array}{c} D_{t}^{\rho }\psi \left(t\right)=\frac{1}{\Gamma (n-\rho )}\int_{0}^{t}{\left(t-\tau \right)}^{n-\rho -1}\psi^{\left(n\right)}\left(t\right)dt, \end{array}$$ (1)

for $n-1<\rho \leq n,\;\;n\in N,\;t>0,\psi \in C_{\lambda }^{n},\;\lambda \geq -1$.

Here, *ρ* and *b* represent the order of the derivative and initial value of the function *ψ*(*t*). Caputo’s fractional derivative has several important properties, some of which are described as follows;

-
$D_{t}^{\rho }A=0,\;\text{where}\;A\;\text{is}\;\text{constant}.$

-
$D_{t}^{\rho }t^{\alpha }=\{\begin{array}{cc} 0, & \text{if}\;\alpha \leq \rho -1,\\ \frac{\Gamma (\alpha +1)}{\Gamma (\alpha -\rho +1)}t^{\alpha -\rho }, & \text{if}\;\alpha >\rho -1. \end{array}$

- Fractional-order differentiations in Caputo’s sense is indeed a linear operation, like to differentiation of integer order:
  $$\begin{array}{c} D_{t}^{\rho }\left(\eta f\left(t\right)+\nu g\left(t\right)\right)=\eta D_{t}^{\rho }f\left(t\right)+\nu D_{t}^{\rho }g\left(t\right), \end{array}$$
  where *η* and *ω* are constants.
- Leibniz rule:
  $$\begin{array}{c} D_{t}^{\rho }\left(f\left(t\right)*g\left(t\right)\right)=\sum_{k=0}^{\infty }\frac{\Gamma (\rho +1)}{\Gamma (k+1)\Gamma (\rho -k+1)}D_{t}^{\rho -k}f\left(t\right)D_{t}^{k}g\left(t\right). \end{array}$$

**Definition 2.3:** The Caputo fractional derivative of order *ρ* > 0 for an integer *n* greater than *ρ*, we can use the following expression:

$$\begin{array}{c} D_{t}^{\rho }\psi \left(x,t\right)=\frac{\partial^{\rho }\psi \left(x,t\right)}{\partial t^{\rho }}=\{\begin{array}{cc} \frac{\partial^{n}\psi \left(x,t\right)}{\partial t^{n}}, & \text{if}\;\rho =n\in N,\\ \frac{1}{\Gamma (n-\rho )}\int_{0}^{t}{\left(t-\tau \right)}^{n-\rho -1}\frac{\partial^{n}\psi \left(x,t\right)}{\partial t^{n}}d\tau , & \text{if}\;n-1<\rho <n. \end{array} \end{array}$$ (2)

To establish our result, it is necessary to introduce the following Riemann-Liouville fractional integral operator.

**Definition 2.4:** The fractional integral whiteSoperator of Riemann Liouville with order *ρ* > 0, of a function *ψ*(*t*) ∈ *C*_λ_, λ ≥ −1, is defined as

aJtρψ(t)=1Γ(ρ)∫at(t-s)ρ-1ψ(s)ds,ρ>0,t>0. (3)

Here, some properties of the operator *J*^*ρ*^ [39], for $\psi \in C_{\lambda },\;\lambda \geq -1,\rho ,\xi >0$ listed as

-
$J_{t}^{0}\psi \left(t\right)]=\psi \left(t\right)$ .
-
$J_{t}^{\rho }J_{t}^{\xi }\psi \left(t\right)=J_{t}^{\rho +\xi }\psi \left(t\right)$ .
-
$J_{t}^{\rho }J_{t}^{\xi }\psi \left(t\right)=J_{t}^{\xi }J_{t}^{\rho }\psi \left(t\right)$ .
-
$J_{t}^{\rho }t^{k}=\frac{\Gamma (k+1)}{\Gamma (\rho +k+1)}t^{\rho +k}$ .

For additional mathematical insights into the properties of fractional integrals and derivatives, interested readers can explore the works cited in [11, 15, 16, 19, 23, 31, 59], as well as the sources referenced within those publications.

## 3 Method description

Let the following be the general fractional order NLEE as

$$\begin{array}{c} D_{t}^{\rho }\psi \left(x,t\right)+\text{R}\psi \left(x,t\right)+\text{N}\psi \left(x,t\right)=h\left(x,t\right), \end{array}$$ (4)

with the following Initial-condition

$$\begin{array}{c} \psi (x,0)=f(x). \end{array}$$ (5)

The Eq (4) can be written as

$$\begin{array}{c} D_{t}^{\rho }\psi \left(x,t\right)=h\left(x,t\right)-\left[N+R\right]\psi \left(x,t\right). \end{array}$$ (6)

Now applying *J*^*ρ*^ to Eq (6), we can obtain

$$\begin{array}{c} \psi \left(x,t\right)=\varphi +J^{\rho }h\left(x,t\right)-J^{\rho }\left(N+R\right)\psi \left(x,t\right), \end{array}$$ (7)

where *φ* is obtain through their initial conditions. The commonly used approach known as the ADM [35, 39, 40] proposes that the solution *μ*(*x*, *t*) can be expressed as an infinite series consisting of components.

$$\begin{array}{c} \psi \left(x,t\right)=\sum_{n=0}^{\infty }\psi_{n}\left(x,t\right), \end{array}$$ (8)

and nonlinear term *Nψ*(*x*, *t*) is decomposed as follows

$$\begin{array}{c} N\psi \left(x,t\right)=\sum_{n=0}^{\infty }A_{n}, \end{array}$$ (9)

where *A*_*n*_ correspond to Adomian polynomials, which are established by the following definition:

$$\begin{array}{c} A_{n}=\frac{1}{n!}{\left[\frac{d^{n}}{d\lambda^{n}}N(\sum_{i=0}^{\infty }\lambda^{i}\psi_{i})\right]}_{\lambda =0},\;\;n\geq 0. \end{array}$$ (10)

For the sake of clarity, the initial several terms of the Adomian polynomials will be provided.

$$\begin{array}{c} \begin{array}{cc} A_{0} & =N(\psi_{0}),\\ A_{1} & =\psi_{1}N^{\left(1\right)}\left(\psi_{0}\right),\\ A_{2} & =\psi_{2}N^{\left(1\right)}\left(\psi_{0}\right)+\frac{1}{2!}\psi_{1}^{2}N^{\left(2\right)}\left(\psi_{0}\right),\\ A_{3} & =\psi_{3}N^{\left(1\right)}\left(\psi_{0}\right)+\psi_{1}\psi_{2}N^{\left(2\right)}\left(\psi_{0}\right)+\frac{1}{3!}\psi_{1}^{3}N^{\left(3\right)}\left(\psi_{0}\right),\\ A_{4} & =\psi_{4}N^{\left(1\right)}\left(\psi_{0}\right)+[\frac{1}{2!}\psi_{2}^{2}+\psi_{1}\psi_{3}]N^{\left(2\right)}\left(\psi_{0}\right)+\frac{1}{2!}\psi_{1}^{2}\psi_{2}N^{\left(3\right)}\left(\psi_{0}\right)+\frac{1}{4!}\psi_{1}^{4}N^{\left(4\right)}\left(\psi_{0}\right),\\ & \vdots \end{array} \end{array}$$ (11)

When we insert the series decompositions Eqs (8) and (9) into both sides of Eq (6), we arrive at the subsequent connection:

$$\begin{array}{c} \sum_{n=0}^{\infty }\psi_{n}\left(x,t\right)=\varphi +J^{\rho }h\left(x,t\right)-J^{\rho }R\sum_{n=0}^{\infty }\psi_{n}\left(x,t\right)-J^{\rho }\sum_{n=0}^{\infty }A_{n}. \end{array}$$ (12)

The Adomian technique is analogous to the subsequent relation, which can be articulated as

$$\begin{array}{cc} \psi_{0}\left(x,t\right) & =\varphi +J^{\rho }h\left(x,t\right),\\ \psi_{1}\left(x,t\right) & =-J^{\rho }[R\psi_{0}+A_{0}],\\ \psi_{2}\left(x,t\right) & =-J^{\rho }[R\psi_{1}+A_{1}],\\ \; & \vdots \\ \psi_{n+1}\left(x,t\right) & =-J^{\rho }[R\psi_{n}+A_{n}]. \end{array}$$

## 4 Fractional order new coupled KdV system

The fractional-order new cKdV system is indeed an intriguing extension of the classical KdV equation. One of the captivating equations within this framework is the fractional-order new cKdV system, expressed as:

$$\begin{array}{c} \begin{array}{cc} D_{t}^{\rho }u & =\beta u_{xxx}+\alpha {\left(uv\right)}_{x}+\gamma {\left(uw\right)}_{x},\\ D_{t}^{\rho }v & =\beta v_{xxx}+\lambda {\left(uw\right)}_{x},\\ D_{t}^{\rho }w & =\beta w_{xxx}+\lambda {\left(uv\right)}_{x}, \end{array} \end{array}$$ (13)

where 0 < *ρ* ≤ 1 and *α*, *β*, *γ*, λ are arbitrary constants, the inclusion of the fractional-order parameter not only extends the classical KdV equation but also prompts a deeper exploration of the system’s behavior. With its fractional derivatives, this system introduces a new dimension to the study of nonlinear wave phenomena. The fractional-order parameter adds complexity, providing a nuanced perspective on the interplay between dispersion and nonlinearity. At *ρ* = 1, this system was investigated by the authors in [42]. This dynamical model delves into the analysis of this fractional-order coupled system, shedding light on its unique dynamics and potential implications for the broader field of nonlinear wave theory.

## 5 Analysis of the technique

In this section, we present the projected fractional IADM as a potent technique for solving the fractional-order new cKdV system. The fractional operator $D_{t}^{\rho }$ represents the Caputo derivative as defined in Eq (1). The method involves applying the inverse operator *J*^*ρ*^ of $D_{t}^{\rho }$ on both sides of Eq (13) to obtain the solution as

$$\begin{array}{c} \begin{array}{cc} u(x,t) & =u\left(x,0\right)+\beta J^{\rho }u_{xxx}+J^{\rho }A,\\ v(x,t) & =v\left(x,0\right)+\beta J^{\rho }v_{xxx}+J^{\rho }B,\\ w(x,t) & =w\left(x,0\right)+\beta J^{\rho }w_{xxx}+J^{\rho }C. \end{array} \end{array}$$ (14)

According to the decomposition technique, we assume that the solution of the unknown functions can be expressed as a series as

$$\begin{array}{c} \begin{array}{c} u\left(x,t\right)=\sum_{n=0}^{\infty }u_{n}\left(x,t\right),\\ v\left(x,t\right)=\sum_{n=0}^{\infty }v_{n}\left(x,t\right),\\ w\left(x,t\right)=\sum_{n=0}^{\infty }w_{n}\left(x,t\right). \end{array} \end{array}$$ (15)

We represented the nonlinear terms in Eq (13) as follows:

$$\begin{array}{c} \begin{array}{c} A=\alpha {\left(uv\right)}_{x}+\gamma {\left(uw\right)}_{x},\\ B=\lambda {\left(uw\right)}_{x},\\ C=\lambda {\left(uv\right)}_{x}, \end{array} \end{array}$$ (16)

where *A*_*n*_, *B*_*n*_, *C*_*n*_ (*n* ≥ 0), denote Adomian polynomials as

$$\begin{array}{c} \begin{array}{c} A_{n}=\frac{1}{n!}\frac{d^{n}}{d\lambda^{n}}N{\left(\sum_{i=0}^{\infty }\lambda^{i}(u\left(x,t\right)\right)}_{\lambda =0},\\ B_{n}=\frac{1}{n!}\frac{d^{n}}{d\lambda^{n}}N{\left(\sum_{i=0}^{\infty }\lambda^{i}(v\left(x,t\right)\right)}_{\lambda =0},\\ C_{n}=\frac{1}{n!}\frac{d^{n}}{d\lambda^{n}}N{\left(\sum_{i=0}^{\infty }\lambda^{i}(w\left(x,t\right)\right)}_{\lambda =0}. \end{array} \end{array}$$ (17)

Substituting the decomposition series Eqs (16) and (15) in Eq (14), we get

$$\begin{array}{c} \begin{array}{c} \sum_{n=0}^{\infty }u_{n}\left(x,t\right)=u\left(x,0\right)+J^{\rho }\beta \sum_{n=0}^{\infty }(u_{n}\left(x,t\right){)}_{xxx}+J^{\rho }\sum_{n=0}^{\infty }A_{n},\\ \sum_{n=0}^{\infty }v_{n}\left(x,t\right)=v\left(x,0\right)+J^{\rho }\beta \sum_{n=0}^{\infty }(v_{n}\left(x,t\right){)}_{xxx}+J^{\rho }\sum_{n=0}^{\infty }B_{n},\\ \sum_{n=0}^{\infty }w_{n}\left(x,t\right)=w\left(x,0\right)+J^{\rho }\beta \sum_{n=0}^{\infty }(w_{n}\left(x,t\right){)}_{xxx}+J^{\rho }\sum_{n=0}^{\infty }C_{n}. \end{array} \end{array}$$ (18)

Hence, through the analysis of decomposition, the subsequent recursive relationships are introduced as

$$\begin{array}{c} \begin{array}{c} u_{0}\left(x,t\right)=u\left(x,0\right).\\ v_{0}\left(x,t\right)=v\left(x,0\right).\\ w_{0}\left(x,t\right)=w\left(x,0\right). \end{array} \end{array}$$ (19)

$$\begin{array}{c} \begin{array}{c} u_{n+1}\left(x,t\right)=J^{\rho }\beta \sum_{n=0}^{\infty }(u_{n}\left(x,t\right){)}_{xxx}+J^{\rho }\sum_{n=0}^{\infty }A_{n}.\\ v_{n+1}\left(x,t\right)=J^{\rho }\beta \sum_{n=0}^{\infty }(v_{n}\left(x,t\right){)}_{xxx}+J^{\rho }\sum_{n=0}^{\infty }B_{n}.\\ w_{n+1}\left(x,t\right)=J^{\rho }\beta \sum_{n=0}^{\infty }(w_{n}\left(x,t\right){)}_{xxx}+J^{\rho }\sum_{n=0}^{\infty }C_{n}. \end{array} \end{array}$$ (20)

Finally, the approximate solution to Eq (13) is obtained through backward substitution as follows:

$$\begin{array}{c} \begin{array}{c} u\left(x,t\right)=u_{0}\left(x,t\right)+u_{1}\left(x,t\right)+u_{2}\left(x,t\right)+....\\ v\left(x,t\right)=v_{0}\left(x,t\right)+v_{1}\left(x,t\right)+v_{2}\left(x,t\right)+....\\ w\left(x,t\right)=w_{0}\left(x,t\right)+w_{1}\left(x,t\right)+w_{2}\left(x,t\right)+.... \end{array} \end{array}$$ (21)

## 6 Numerical results

Consider fractional order new coupled KdV system in Eq (13) with *ϵ* = 2, *K* = 1, *d*_2_ = −2.

For the sake of simulation, we also include the exact solution of the fractional new cKdV system from Eq (13), established in reference [42] for *ρ* = 1, as follows

$$\begin{array}{l} u(x,t)=\frac{2\beta d_{2}K^{2}}{3\lambda }\left(2-\frac{3+{\text{tanh}}^{2}\left(\varepsilon \sqrt{-\frac{d_{2}}{3}}(Kx-\omega t)\right)}{{\text{tanh}}^{2}\left(\varepsilon \sqrt{-\frac{d_{2}}{3}}(Kx-\omega t)\right)}\right),\\ v(x,t)=-\frac{\beta d_{2}K^{2}(\alpha -\sqrt{\alpha^{2}+4\gamma \lambda }}{3\lambda }\left(2-\frac{3+{\text{tanh}}^{2}\left(\varepsilon \sqrt{-\frac{d_{2}}{3}}(Kx-\omega t)\right)}{{\text{tanh}}^{2}\left(\varepsilon \sqrt{-\frac{d_{2}}{3}}(Kx-\omega t)\right)}\right),\\ w(x,t)=\frac{\beta h_{2}K^{2}(\alpha -\sqrt{\alpha^{2}+4\gamma \lambda }}{3\lambda }\left(2-\frac{3+{\text{tanh}}^{2}\left(\varepsilon \sqrt{-\frac{d_{2}}{3}}(Kx-\omega t)\right)}{{\text{tanh}}^{2}\left(\varepsilon \sqrt{-\frac{d_{2}}{3}}(Kx-\omega t)\right)}\right), \end{array}$$ (22)

where $\omega =\pm \frac{4\beta d_{2}K^{2}}{3}$. Now, let’s consider the initial condition at *t* = 0 as stated in Eq (22), and investigate the following two cases as

$$\begin{array}{l} u(x,0)=\frac{2\beta d_{2}K^{2}}{3\lambda }\left(2-\frac{3+{\text{tanh}}^{2}\left(\varepsilon \sqrt{-\frac{d_{2}}{3}}Kx\right)}{{\text{tanh}}^{2}\left(\varepsilon \sqrt{-\frac{d_{2}}{3}}Kx\right)}\right).\\ v(x,0)=-\frac{\beta d_{2}K^{2}(\alpha -\sqrt{\alpha^{2}+4\gamma \lambda }}{3\lambda }\left(2-\frac{3+{\text{tanh}}^{2}\left(\varepsilon \sqrt{-\frac{d_{2}}{3}}Kx\right)}{{\text{tanh}}^{2}\left(\varepsilon \sqrt{-\frac{d_{2}}{3}}Kx\right)}\right).\\ w(x,0)=\frac{\beta d_{2}K^{2}(\alpha -\sqrt{\alpha^{2}+4\gamma \lambda }}{3\lambda }\left(2-\frac{3+{\text{tanh}}^{2}\left(\varepsilon \sqrt{-\frac{d_{2}}{3}}Kx\right)}{{\text{tanh}}^{2}\left(\varepsilon \sqrt{-\frac{d_{2}}{3}}Kx\right)}\right). \end{array}$$ (23)

**Case 1:** *α* = 0.001, *β* = 0.001, λ = 0.01, *γ* = 0.01, *ϵ* = 2.

**Case 2:** *α* = 0.01, *β* = 0.01, λ = 0.1, *γ* = 0.1, *ϵ* = 1.5.

The simulation results for the two aforementioned cases are now presented. Tables 1 through 3 display an analysis of different combinations of *x* and *t*, comparing these outcomes with the exact solution obtained for Case 1. These tables provide a comprehensive view of the system’s behavior at different spatial and temporal points. In Tables 4 through 6, we similarly explore various values of *x* and *t*, but focus on the simulated results derived from the exact solution for Case 2. This comparative analysis allows us to assess the accuracy and performance of the numerical approximations under different parameter configurations. These tables include values for the exact solution, as well as the approximate solutions for two diverse values of *ρ* (1 and 0.5). The calculated error values offer insights into the convergence and reliability of the numerical simulations.

**Table 1:** Approximate solution (*n* = 2) is computed of *u*(*x*, *t*) for various values of *ρ*, and a comparison of the absolute error is performed at *ρ* = 1 of Eq (21) for Case 1.

*t*	*x*	Exact solution	Approximate Solution *ρ* = 1	Approximate Solution *ρ* = 0.5	Absolute Error
0.1	1	0.33260008414074643	0.33242137089454	0.33242137089454	1.7871324620644113 × 10^−4^
0.2	2	0.26899919711886017	0.2689933891861401	0.2689933891861401	5.807932720092168 × 10^−6^
0.3	3	0.26675534834141734	0.2667550306812397	0.2667550306812397	3.176601776622156 × 10^−7^
0.4	4	0.26667004738901595	0.26667003131216377	0.26667003131216377	1.607685218285581 × 10^−8^
0.5	5	0.2666667955597643	0.2666667947958156	0.2666667947958156	7.639486820920638 × 10^−10^
0.6	6	0.2666666715808518	0.2666666715460007	0.2666666715460007	3.485106647715952 × 10^−11^
0.7	7	0.2666666668540253	0.26666666685247964	0.26666666685247964	1.545652494883143 × 10^−12^
0.8	8	0.2666666666738099	0.2666666666737428	0.2666666666737428	6.711298183859071 × 10^−14^
0.9	9	0.2666666666669389	0.26666666666693606	0.26666666666693606	2.831068712794149 × 10^−15^
1	10	0.2666666666666772	0.2666666666666769	0.2666666666666769	3.330669073875469 × 10^−16^

**Table 2:** Approximate solution (*n* = 2) is computed of *v*(*x*, *t*) for various values of *ρ*, and a comparison of the absolute error is performed at *ρ* = 1 of Eq (21) for Case 1.

*t*	*x*	Exact solution	Approximate Solution *ρ* = 1	Approximate Solution *ρ* = 0.5	Absolute Error
0.1	1	0.31638557051936744	0.31624294367981326	0.31624294367981326	1.4262683955418298 × 10^−4^
0.2	2	0.25588527636597774	0.2558812220386966	0.2558812220386966	4.054327281122205 × 10^−6^
0.3	3	0.25375081696725427	0.2537505973519397	0.2537505973519397	2.19615314545063 × 10^−7^
0.4	4	0.2536696745028406	0.25366966338180913	0.25366966338180913	1.112103148459553 × 10^−8^
0.5	5	0.2536665812028024	0.253666580673836	0.253666580673836	5.289663707053194 × 10^−10^
0.6	6	0.2536664632679585	0.2536664632438035	0.2536664632438035	2.415501132446707 × 10^−11^
0.7	7	0.2536664587715685	0.2536664587704962	0.2536664587704962	1.072308908334207 × 10^−12^
0.8	8	0.25366645860013876	0.2536664586000922	0.2536664586000922	4.657385588302532 × 10^−14^
0.9	9	0.25366645859360276	0.2536664585936008	0.2536664585936008	1.942890293094024 × 10^−15^
1	10	0.2536664585933538	0.25366645859335357	0.25366645859335357	2.220446049250313 × 10^−16^

**Table 3:** Approximate solution (*n* = 2) is computed of *w*(*x*, *t*) for various values of *ρ*, and a comparison of the absolute error is performed at *ρ* = 1 of Eq (21) for Case 1.

*t*	*x*	Exact solution	Approximate Solution *ρ* = 1	Approximate Solution *ρ* = 0.5	Absolute Error
0.1	1	−0.31638557051936744	−0.31624294367981326	−0.31624294367981326	1.4262683955418298 × 10^−4^
0.2	2	−0.25588527636597774	−0.2558812220386966	−0.2558812220386966	4.054327281122205 × 10^−6^
0.3	3	−0.25375081696725427	−0.2537505973519397	−0.2537505973519397	2.19615314545063 × 10^−7^
0.4	4	−0.2536696745028406	−0.25366966338180913	−0.25366966338180913	1.112103148459553 × 10^−8^
0.5	5	−0.2536665812028024	−0.253666580673836	−0.253666580673836	5.289663707053194 × 10^−10^
0.6	6	−0.2536664632679585	−0.2536664632438035	−0.2536664632438035	2.415501132446707 × 10^−11^
0.7	7	−0.2536664587715685	−0.2536664587704962	−0.2536664587704962	1.072308908334207 × 10^−12^
0.8	8	−0.25366645860013876	−0.2536664586000922	−0.2536664586000922	4.657385588302532 × 10^−14^
0.9	9	−0.25366645859360276	−0.2536664585936008	−0.2536664585936008	1.942890293094024 × 10^−15^
1	10	−0.2536664585933538	−0.25366645859335357	−0.25366645859335357	2.220446049250313 × 10^−16^

**Table 4:** Approximate solution (*n* = 2) is computed of *u*(*x*, *t*) for various values of *ρ*, and a comparison of the absolute error is performed at *ρ* = 1 of Eq (21) for Case 2.

*t*	*x*	Exact solution	Approximate Solution *ρ* = 1	Approximate Solution *ρ* = 0.5	Absolute Error
0.1	1	0.33204482166407284	0.33069799422511365	0.3301655580378987	1.3468274389591972 × 10^−3^
0.2	2	0.26896281180421	0.268909716688929	0.2687898625861433	5.3095115281009964 × 10^−5^
0.3	3	0.2667532870873321	0.26675043269263665	0.26674582304681194	2.854394695439577 × 10^−6^
0.4	4	0.26666994303567765	0.2666698038643308	0.26666964007414545	1.391713468357203 × 10^−7^
0.5	5	0.2666667906059158	0.2666667842538761	0.26666677865788957	6.352039727541836 × 10^−9^
0.6	6	0.2666666713550871	0.2666666710773379	0.26666667089232227	2.7774921251833 × 10^−10^
0.7	7	0.266666666844022	0.26666666683224133	0.2666666668263283	1.178068753659999 × 10^−11^
0.8	8	0.26666666667337585	0.26666666667288746	0.2666666666727059	4.883871085326064 × 10^−13^
0.9	9	0.26666666666692057	0.26666666666690053	0.2666666666668952	2.003952559448407 × 10^−14^
1	10	0.2666666666666762	0.26666666666667543	0.26666666666667527	7.771561172376096 × 10^−16^

**Table 5:** Approximate solution (*n* = 2) is computed of *v*(*x*, *t*) for various values of *ρ*, and a comparison of the absolute error is performed at *ρ* = 1 of Eq (21) for Case 2.

*t*	*x*	Exact solution	Approximate Solution *ρ* = 1	Approximate Solution *ρ* = 0.5	Absolute Error
0.1	1	0.31585737752168913	0.31479254701256393	0.31425718876913666	1.064830509125203 × 10^−3^
0.2	2	0.2558506648638074	0.2558130690623424	0.25571106346062705	3.759580146500685 × 10^−5^
0.3	3	0.2537488562009141	0.25374682575849444	0.2537428251638899	2.030442419642675 × 10^−6^
0.4	4	0.25366957523680905	0.25366947505338366	0.2536693297053994	1.001834253888667 × 10^−7^
0.5	5	0.2536665764904579	0.25366657185808894	0.25366656677559024	4.632368932888653 × 10^−9^
0.6	6	0.2536664630532001	0.25366646284778355	0.2536664626755915	2.054165726406154 × 10^−10^
0.7	7	0.253666458762053	0.253666458753207	0.2536664587475591	8.846035015608322 × 10^−12^
0.8	8	0.2536664585997259	0.2536664585993531	0.2536664585991748	3.728128916691275 × 10^−13^
0.9	9	0.2536664585935854	0.25366645859356973	0.2536664585935644	1.56541446472147 × 10^−14^
1	10	0.25366645859335296	0.25366645859335235	0.2536664585933522	6.106226635438361 × 10^−16^

**Table 6:** Approximate solution (*n* = 2) is computed of *w*(*x*, *t*) for various values of *ρ*, and a comparison of the absolute error is performed at *ρ* = 1 of Eq (21) for Case 2.

*t*	*x*	Exact solution	Approximate Solution *ρ* = 1	Approximate Solution *ρ* = 0.5	Absolute Error
0.1	1	−0.31585737752168913	−0.31479254701256393	−0.31425718876913666	1.064830509125203 × 10^−3^
0.2	2	−0.2558506648638074	−0.2558130690623424	−0.25571106346062705	3.759580146500685 × 10^−5^
0.3	3	−0.2537488562009141	−0.25374682575849444	−0.2537428251638899	2, 030442419642675 × 10^−6^
0.4	4	−0.25366957523680905	−0.25366947505338366	−0.2536693297053994	1.001834253888667 × 10^−7^
0.5	5	−0.2536665764904579	−0.25366657185808894	−0.25366656677559024	4.632368932888653 × 10^−9^
0.6	6	−0.2536664630532001	−0.25366646284778355	−0.2536664626755915	2.054165726406154 × 10^−10^
0.7	7	−0.253666458762053	−0.253666458753207	−0.2536664587475591	8.846035015608322 × 10^−12^
0.8	8	−0.2536664585997259	−0.2536664585993531	−0.2536664585991748	3.728128916691275 × 10^−13^
0.9	9	−0.2536664585935854	−0.25366645859356973	−0.2536664585935644	1.56541446472147 × 10^−14^
1	10	−0.25366645859335296	−0.25366645859335235	−0.2536664585933522	6.106226635438361 × 10^−16^

**Fig 1:**
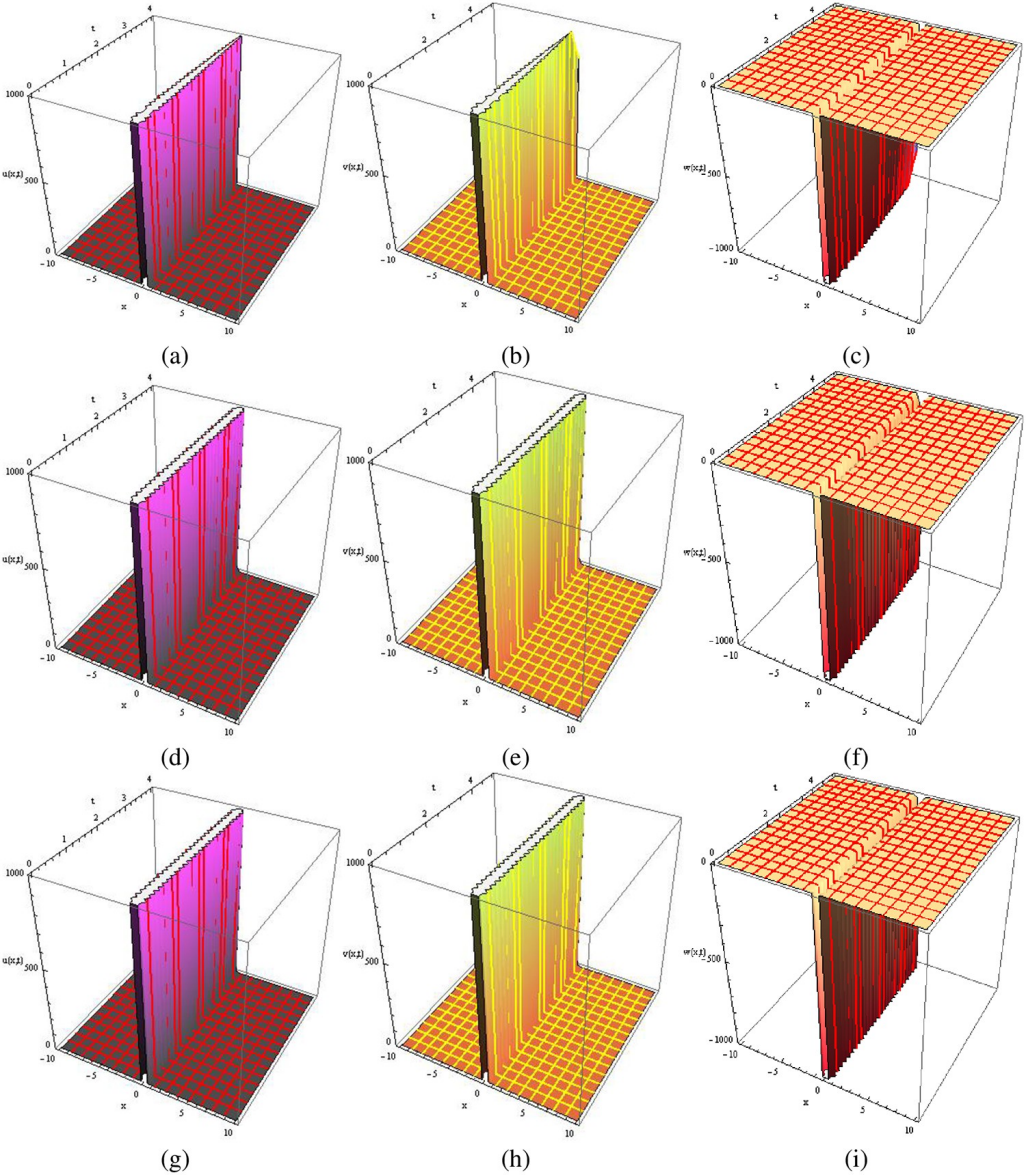
The 3D graphical comparison of exact solutions ((a), (b), (c) at *ρ* = 1) with approximations ((d), (e), (f) at *ρ* = 1, and (g), (h), (i) at *ρ* = 0.5) for *u*, *v*, and *w* in Case 1.

**Fig 2:**
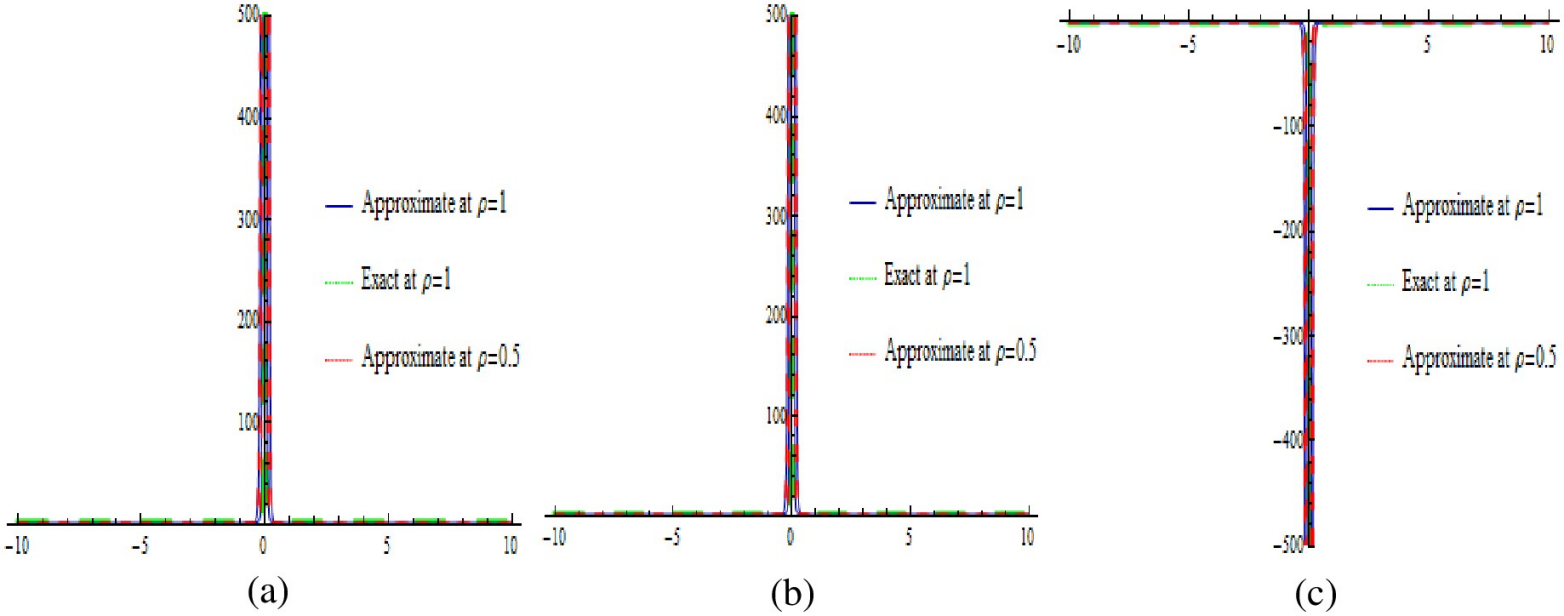
The 2D graphical comparison of exact and approximate solutions for Case 1.

**Fig 3:**
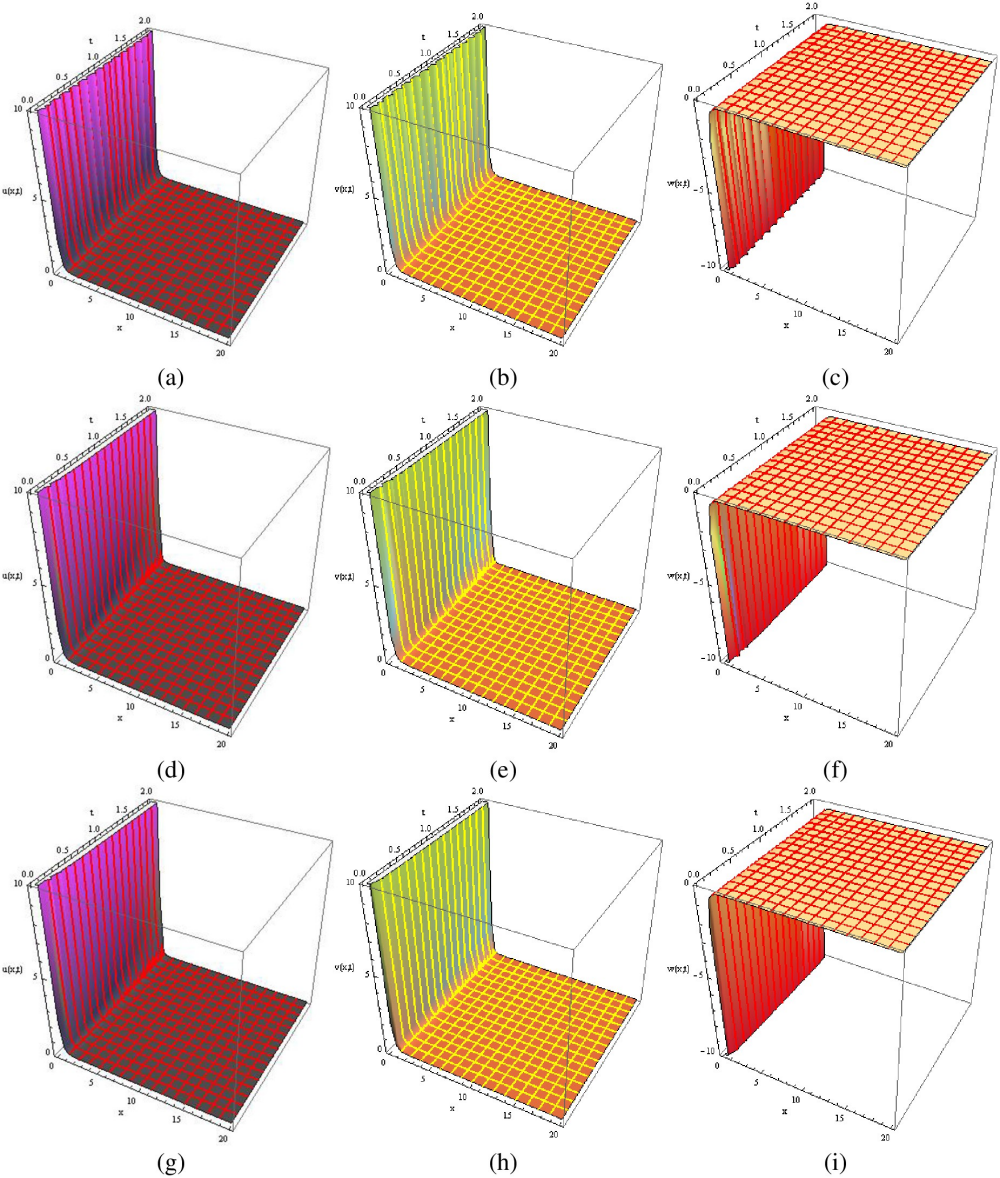
The 3D graphical comparison of exact solutions ((a), (b), (c) at *ρ* = 1) with approximations ((d), (e), (f) at *ρ* = 1, and (g), (h), (i) at *ρ* = 0.5) for *u*, *v*, and *w* in Case 2.

**Fig 4:**
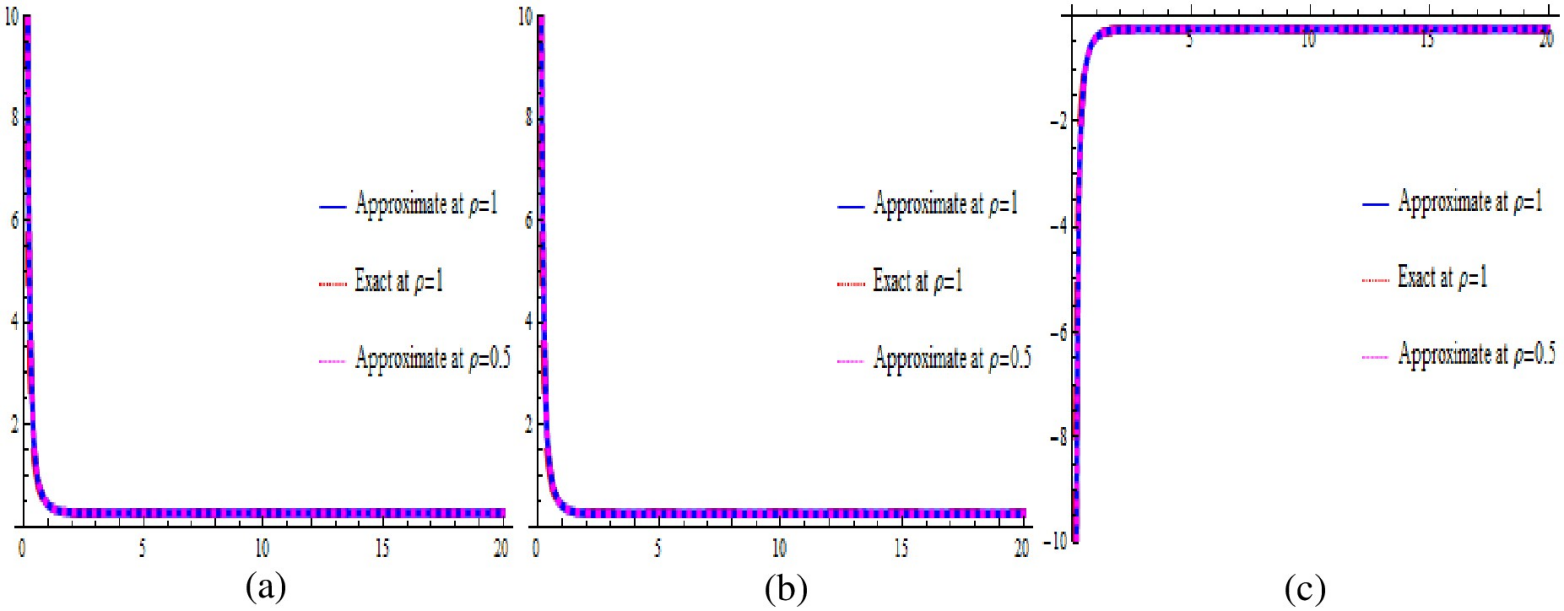
The 2D graphical comparison of exact and approximate solutions for Case 2.

Visual representations of the solutions to the fractional-order new cKdV system for the two considered cases are showcased in Figs 1–4. Specifically, Figs 1 and 3 present three-dimensional profiles capturing the exact solution (at *ρ* = 1), approximate solution (at *ρ* = 1) and approximate solution (at *ρ* = 0.5) of the system described by Eq (13). These 3D graphs offer an insightful depiction of the system’s behavior under varying parameter configurations. In contrast, Figs 2 and 4 provide two-dimensional representations, offering a focused view of specific aspects of the solutions. In each case, the exact solution in Eq (22). is juxtaposed with the corresponding numerical solution obtained from Eq (21). This side-by-side presentation facilitates a visual assessment of the accuracy and agreement between the analytical predictions and the results derived from numerical simulations, encompassing both 2D and 3D graphs. The color-coded surfaces in each figure emphasize the dynamic nature of the solutions, allowing for a more intuitive understanding of the system’s response in each case.

## 7 Conclusion

In this work, we successfully employed the projected fractional improved ADM to the fractional-order new cKdV system and obtained approximate solutions, thereby extending its applicability to phenomena with non-integer order characteristics and spatial extensions. The fractional new cKdV system extends the classical coupled KdV system, incorporating fractional calculus for a more accurate representation of wave interactions, which finds applications in various areas such as fluid dynamics, plasma physics, nonlinear optics, and other fields of mathematical physics. This enhanced model promises to deepen our understanding and predictive capabilities across these disciplines, paving the way for advancements in theoretical and applied research. This technique is significantly recognized for its effectiveness in addressing nonlinearities and iteratively handling fractional derivatives. These solutions play a crucial role in designing and optimizing systems in engineering applications where accurate modeling of wave phenomena is essential, including fluid dynamics, plasma physics, nonlinear optics, and other mathematical physics domains. Comparison with exact solutions demonstrates the high accuracy and ease of application of the projected method. The numerical experiments conducted in this study serve a dual purpose: validating the accuracy and efficiency of the ADM-based approach and unraveling the intricacies associated with fractional-order systems. These findings significantly contribute to our understanding of the behavior of fractional-order KdV systems. The obtained approximate results prove that this proposed technique is influential in solving fractional coupled systems encountered in various fields such as engineering and physics. Numerical results obtained using Mathematica software further verify and demonstrate its efficacy.
